# Tripodal Triptycenes as a Versatile Building Block for Highly Ordered Molecular Films and Self-Assembled Monolayers

**DOI:** 10.1021/acs.accounts.4c00743

**Published:** 2025-01-07

**Authors:** Michael Zharnikov, Yoshiaki Shoji, Takanori Fukushima

**Affiliations:** † Applied Physical Chemistry, Heidelberg University, Im Neuenheimer Feld 253, D-69120 Heidelberg, Germany; ‡ Laboratory for Chemistry and Life Science (CLS), Institute of Integrated Research, Institute of Science Tokyo, 4259 Nagatsuta, Midori-ku, Yokohama 226-8501, Japan; § Research Center for Autonomous Systems Materialogy (ASMat), Institute of Integrated Research, Institute of Science Tokyo, 4259 Nagatsuta, Midori-ku, Yokohama 226-8501, Japan

## Abstract

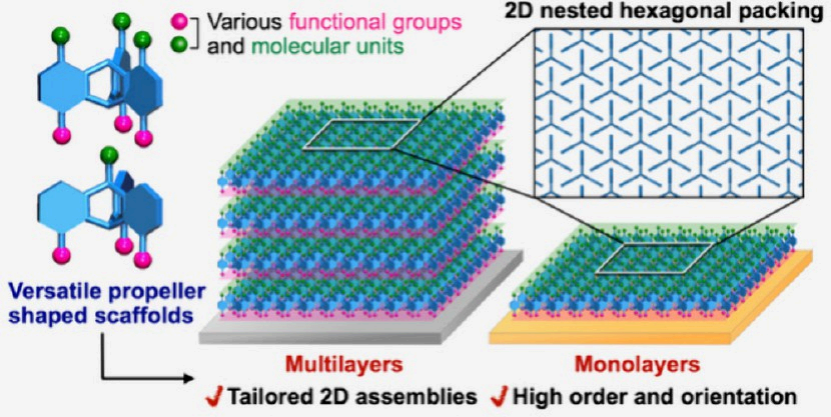

The design of properties and functions of molecular assemblies requires not only a proper choice of building blocks but also control over their packing arrangements. A highly versatile unit in this context is a particular type of triptycene with substituents at the 1,8,13-positions, called tripodal triptycene, which offers predictable molecular packing and multiple functionalization sites, both at the opposite 4,5,16- or 10 (bridgehead)-positions. These triptycene building blocks are capable of two-dimensional (2D) nested hexagonal packing, leading to the formation of 2D sheets, which undergo one-dimensional (1D) stacking into well-defined “2D+1D” structures. This ability makes it possible to form large-area molecular films having long-range structural integrity even on polymer substrates, which can be used to enhance the performance of organic devices. Importantly, the 2D assembly ability of tripodal triptycenes is robust and not impaired when chemically modified with functional molecular units and even with polymer chains. In addition, introducing suitable functionalities that act as anchoring groups results in reliable tripodal monomolecular assembly on application-relevant inorganic substrates, which is generally considered quite a challenging task. Self-assembled monolayers (SAMs) have been formed on Au(111), Ag(111), and indium tin oxide. On gold, these SAMs feature the nested hexagonal packing typical of 2D triptycene sheets, whereas, on silver, a distinct polymorphism with several different packing motifs occurs. Along with basic, nonsubstituted tripodal SAMs, specifically functionalized monolayers have been designed. A substitution pattern in which three nitrile tail groups build the outermost surface of a tripodal triptycene-based SAM has allowed for the study of femtosecond charge transfer dynamics across the triptycene framework, with a particular emphasis on the so-called matrix effects involving intramolecular pathways. The functionalization of the bridgehead position with a ferrocene tail group has enabled single-molecule observation of redox reactions and the creation of assemblies of unique molecular rectifiers, exhibiting highly effective rectification at a very low bias voltage. Complementary to the synthesis of these complex functional triptycenes, a strategy of on-surface click reactions has been designed. Indeed, a tripodal triptycene having an ethynyl tail group at the 10-position, capable of click reactions with azide functionalities, works well, allowing successive molecular layer deposition. The performance of tripodal triptycene-based SAMs has also been tested in the context of electron beam lithography (EBL) and nanofabrication, leading to the finding that these SAMs can serve as negative resists for EBL due to the efficient cross-linking, giving rise to triptycene-stemming carbon nanomembranes (CNM). These membranes feature the lowest lateral material densities used to date for CNM preparation, which makes them unique in this regard.

## Key References

- SeikiN.; ShojiY.; KajitaniT.; IshiwariF.; KosakaA.; HikimaT.; TakataM.; SomeyaT.; FukushimaT.Rational synthesis of organic thin films with exceptional long-range structural integrity. Science2015, 348, 1122–1126.26045433 10.1126/science.aab1391(1) A space-filling design, relying on the nested hexagonal packing of a particular type of triptycene, enables the formation of molecular films with long-range 2D structural integrity.
- LeungF. K.-C.; IshiwariF.; KajitaniT.; ShojiY.; HikimaT.; TakataM.; SaekiA.; SekiS.; YamadaY. M. A.; FukushimaT.Supramolecular scaffold for tailoring the two-dimensional assembly of functional molecular units into organic thin films. J. Am. Chem. Soc.2016, 138, 11727–11733.27549349 10.1021/jacs.6b05513(2) Bridgehead-substituted triptycenes provide a versatile supramolecular scaffold for tailoring the 2D assembly of molecular units into a highly oriented thin film.
- IshiwariF.; NascimbeniG.; SauterE.; TagoH.; ShojiY.; FujiiS.; KiguchiM.; TadaT.; ZharnikovM.; ZojerE.; FukushimaT.Triptycene Tripods for the Formation of Highly Uniform and Densely Packed Self-Assembled Monolayers with Controlled Molecular Orientation. J. Am. Chem. Soc.2019, 141, 5995–6005.30869881 10.1021/jacs.9b00950PMC6483319(3) High-quality tripodal-anchored self-assembled monolayers on Au(111) are formed.
- DasS.; IshiwariF.; ShojiY.; FukushimaT.; ZharnikovM.Triptycene-Based Self-Assembled Monolayer as a Template for Successive Click Reactions. J. Phys. Chem. C2023, 127, 5178–5185.(4) Decoration of triptycene-based self-assembled monolayers with different substituents can be performed by on-surface click reactions.

## Introduction

**Figure 1:**
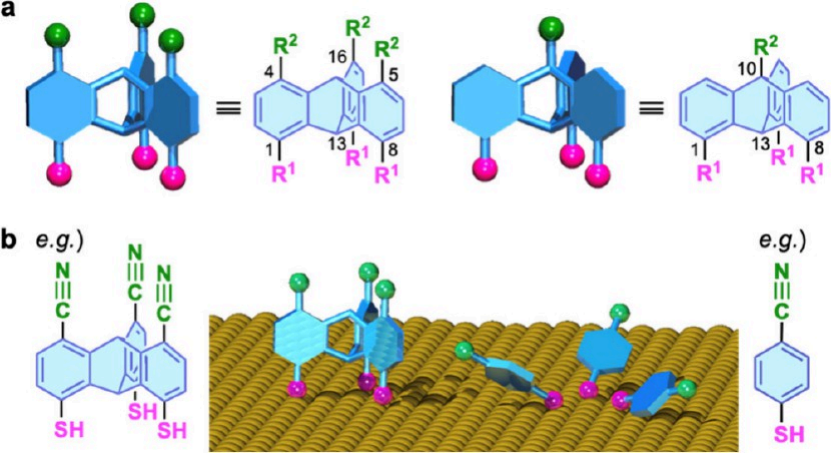
(a) Schematic illustration of 1,8,13-substituted tripodal triptycene derivatives having substituents at the 4,5,16-positions (left) and bridgehead 10-position (right) with the carbon numbering for triptycene. (b) Schematic illustration of tripodal anchoring (left) and monoanchoring (right) systems on a solid support, with the tail and anchoring groups drawn in green and pink, respectively.

**Figure 2:**
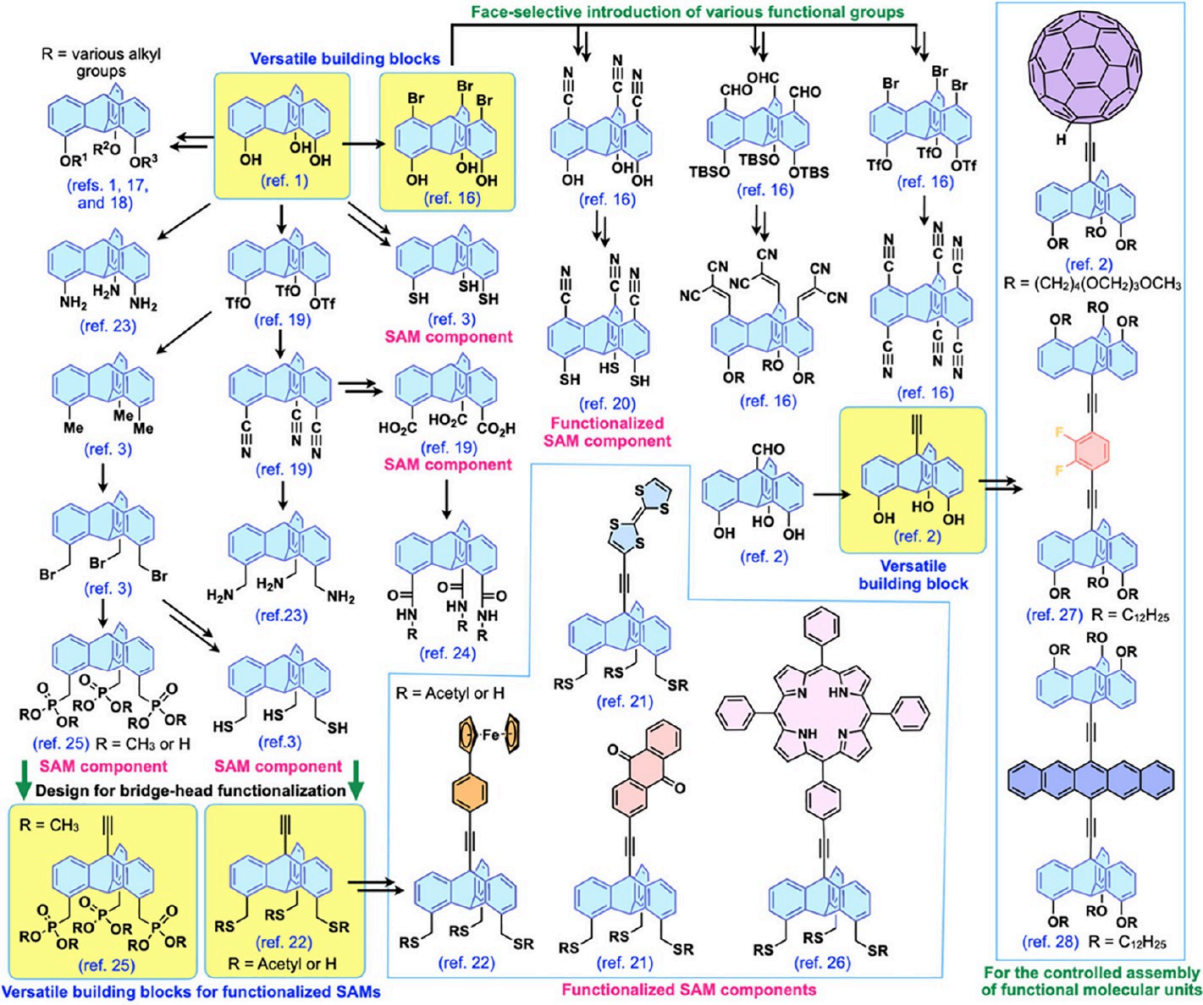
Examples of triptycene derivatives we have developed so far. The corresponding reference numbers are shown in parentheses.

Control over the molecular arrangement and orientation in organic thin films is important for developing the properties of these constructs and the performance of devices using them as functional components. However, in most cases, it is difficult to predict the assembled forms from the structures of the constituent molecules. This can, however, be achieved by the use of covalent or noncovalent postfunctionalization of molecular building blocks that exhibit a “robust” self-assembling ability, forming predictable and well-defined structures.^5^ A promising unit in this regard is triptycene (Figure 1a),^6,7^ which has a rigid propeller-shaped backbone consisting of three 120°-oriented phenyl rings connected by aliphatic bridges. The 1,8,13-substituted form of triptycene is capable of nested hexagonal packing of its phenylene blades, leading to the formation of 2D sheets, which undergo one-dimensional (1D) stacking, resulting in ordered 2D+1D structures. Taking advantage of the design flexibly in substitution pattern through the attachment of functional groups at the opposite tripodal 4,5,16-positions or the bridgehead 10-position of the triptycene backbone (Figure 1a), it is possible to create a wide variety of building blocks for the fabrication of highly organized multilayer and monolayer films on solid supports. In the former case, we have achieved high structural integrity in a size regime beyond the micrometer length scale. This is a difficult task since organic thin films are usually formed by nucleation of individual domains that propagate and merge, resulting in small-sized microdomain structures. The multilayer films are weakly coupled to the substrate since the assembly is driven entirely by intermolecular forces (apart from the lateral confinement imposed by the substrate). Adjusting these forces so that individual triptycene-based building blocks can undergo controlled assembly into the desired 2D+1D structures even when other molecular units are incorporated is important. On the other hand, when introducing specific groups capable of chemical bonding to the solid surface, monolayer films (*i.e*., SAMs) coupled strongly to the solid support, can be constructed. In this case, anchoring groups can be selected according to their affinity to the target substrate while it is also desired to design the opposite face of the triptycene moiety to avoid covalent bonding to the substrate as well as the formation of multilayers. In general, SAMs are constructed using rod-like molecules bearing an anchoring group, responsible for bonding to the substrate, and a functional tail group, exposed to an ambient environment.^8−11^ Particular advantages of tripodal triptycenes for SAM formation over monoanchoring systems include their ability to achieve stronger bonding and better electronic coupling to the substrate, better control over the orientation of the tail groups, tuning of their packing density, and additional possibilities of structural arrangements (Figure 1b). Note that reliable tripodal bonding of SAM-forming molecules has, however, scarcely been achieved so far, with the consequence that structural uniformity and orientational order in tripodal SAMs are usually poor.^12−15^ We expected that the triptycene scaffold, in contrast to the tetrahedral cores most commonly used for tripodal assemblies,^12−15^ would allow us to overcome this obstacle, which has turned out to be true, as will be discussed below. In this Account, we exclusively focus on the design, assembly, and functional properties of tripodal triptycene-based molecules and SAM precursors. Although the synthesis of these compounds is described in detail in the original publications, we here summarize examples of the various triptycene derivatives we have developed in Figure 2.^1−3,16−28^ Synthetic aspects concerning functional triptycene-based molecules have also been discussed in recent reviews including those by Shindo et al., Kaleta et al., and our group,^29−31^ the latter two of which also describe fundamental physical properties and illustrate specific functional abilities of these triptycene derivatives.

## Long-Range Ordered Multilayer Films

**Figure 3:**
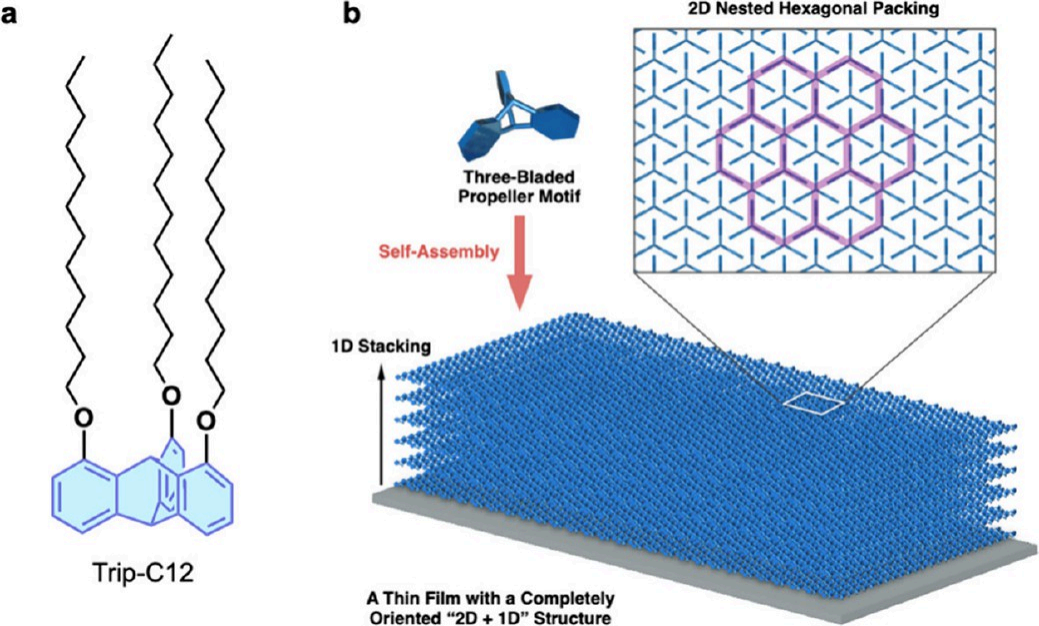
Schematics of (a) the chemical structure of a representative paraffinic triptycene molecule, Trip-C12, and (b) a “2D (hexagonal triptycene array) + 1D (layer stacking)” structure of multilayer films of tripodal triptycenes. Adapted with permission from ref (1). Copyright 2015 American Association for the Advancement of Science.

As mentioned in the Introduction, it is difficult to construct organic thin films with long-range molecular ordering in a size regime beyond the micrometer length scale. Inspired by the structure of highly oriented pyrolytic graphite, we solved this problem by designing a 2D hexagonal array of tripodal triptycenes, which stack up to form a 2D+1D structure.^1^Figure 3a shows a typical example of such assemblies formed from tripodal paraffinic triptycenes (Trip-C12). These triptycenes serve as fundamental building blocks to achieve nested hexagonal packing in individual 2D arrays (Figure 3b). Completely oriented multilayered assemblies of Trip-C12 featuring a 2D+1D structure (Figure 3c) can then be grown by vacuum evaporation, spin-coating, or cooling from the isotropic liquid of the triptycene, resulting in organic thin films with exceptional structural order. According to the X-ray diffraction (XRD) and grazing incidence XRD (GI-XRD) data, these films exhibit long-range 2D structural integrity up to the centimeter length scale. It is assumed that, in contrast to usual molecular films, the uniform assembly of Trip-C12 does not evolve from locally occurring nucleation sites but rather via fusion of dynamic domains, wherein the triptycene molecules, capable of nested packing, fluctuate concertedly in such a way as to correct lattice mismatches.

The triptycene films can be formed with the same quality on different substrates, which is understandable given their weak coupling with the substrate, so its type is of minor importance. They are also sufficiently robust to preserve their structure up to a temperature close to the melting point of the constituent molecules (*e*.*g*., 210 °C for Trip-C12). Triptycene derivatives with different alkoxy chain lengths as well as those with one or two long-chain alkoxy groups also form 2D+1D structures similar to that of Trip-C12.^1,31^ It is essential to choose appropriate substituents and substitution patterns to realize such structures, considering the fact that nonsubstituted triptycene does not show nested hexagonal packing in the crystal or on substrate surfaces.^32,33^

## Supramolecular Scaffolds

**Figure 4:**
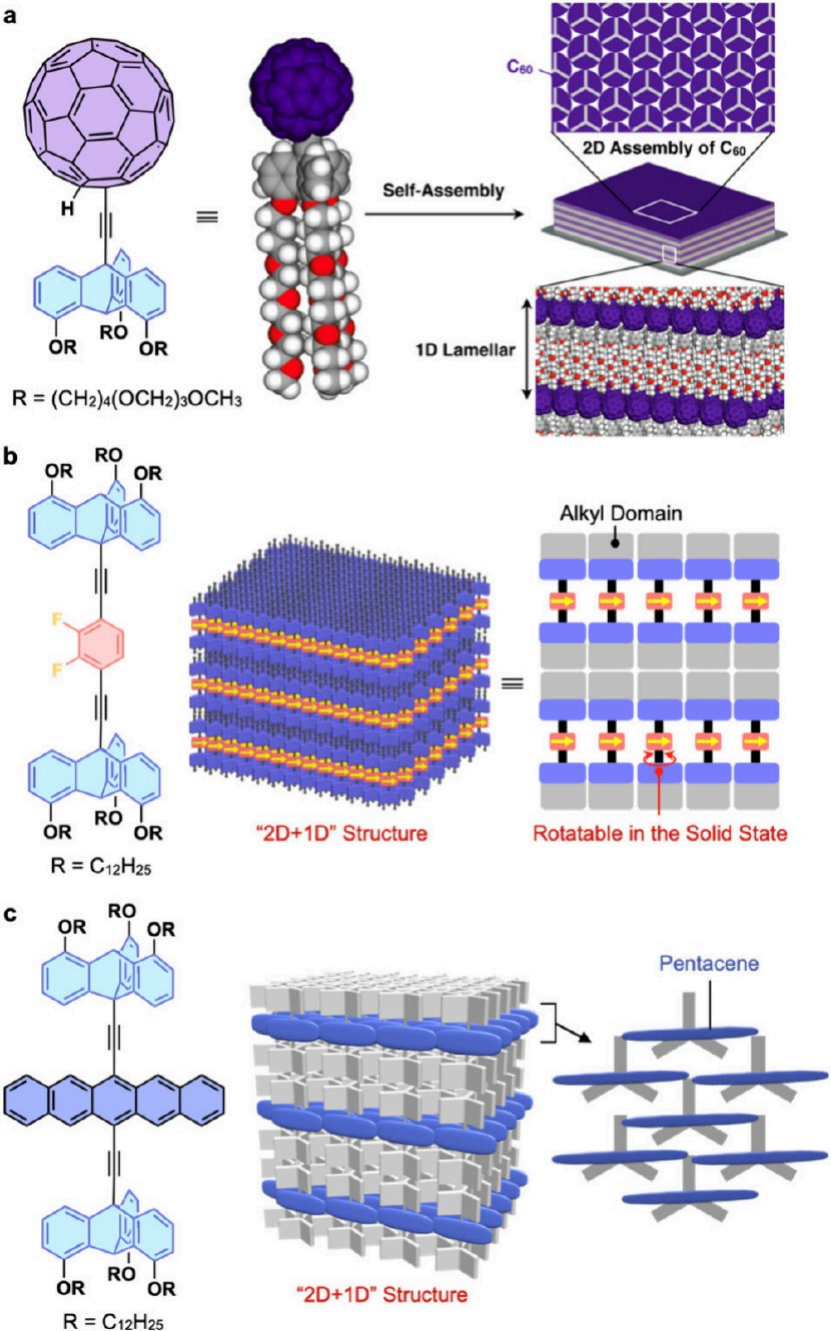
Schematics of molecular and assembly structures of (a) C_60_-appended, (b) molecular rotor-appended, and (c) pentacene-appended tripodal triptycene derivatives. Adapted with permission from refs (a) (2), (b) (27), and (c) (28). Copyrights 2016 American Chemical Society, 2024 Royal Society of Chemistry, and 2024 The Authors, available under a CC BY 4.0 license.

Highly ordered, anisotropic triptycene assemblies are of interest not only by themselves but also of use as supramolecular scaffolds^5^ to tailor 2D assemblies of functional molecular units into thin film forms. Figure 4 illustrates three examples. In the first case, tripodal triptycenes, to which a C_60_ unit is attached through the bridge-head ethynyl group, were used (Figure 4a).^2^ Surprisingly, these molecules form a similar “2D hexagonal +1D lamellar” structure to the parent paraffinic triptycenes (Trip-C12), with the C_60_ units densely packed in a 2D fashion. This structure exhibits highly anisotropic charge-transport properties, where the transient conductivity within the 2D layer is 5.3 times greater than interlayer one.^2^

The second example is the assembly of a molecular rotor using a triptycene-based scaffold.^27^ The designed molecule consists of a dipolar 1,2-difluorobenzene rotor sandwiched by two 10-ethynyl-1,8,13-tridodecyloxy triptycenes (Figure 4b) and self-assembles to form a 2D+1D structure. Importantly, the dipolar rotor units align in a 2D fashion with a spacing of ∼0.8 nm, providing sufficient space for rotational motion. Solid-state ^19^F NMR spectroscopic analysis suggests that the rotor units on the triptycene scaffold exhibit kHz-order motion with an activation energy of approximately 5.7 kcal mol^–1^.^27^

The third example provides a molecular design concept to achieve efficient singlet fission (SF) of acene chromophores. A sandwich-type molecule with a pentacene unit was designed, which self-assembles to form a 2D+1D structure upon solution casting onto a solid substrate (Figure 4c).^28^ In this assembly, the pentacene units are arranged two-dimensionally with sufficient overlap to cause SF, while enough space is left to allow for conformational change of the chromophore, facilitating the dissociation of the generated triplet pairs into free triplets. Due to these structural features, a cast film of this assembly exhibits rapid SF with a rate constant of 5.9 × 10^12^ s^–1^ as well as efficient generation of triplet pairs with a quantum yield of 88 ± 5%, as revealed by femtosecond transient absorption spectroscopy. Furthermore, the generated triplet pairs undergo dissociation into free triplets with a quantum yield of 130 ± 8.8%. Note that an analogous sandwich-type triptycene derivative having an anthracene unit also self-assembles to form a 2D+1D structure, however, no SF is observed.^28^ This is because there is no effective overlap of the smaller anthracene units in the assembly. The observed difference between these pentacene and anthracene systems suggests that the triptycene-based scaffold enables precise arrangement of the incorporated functional molecular units at prescribed distances allowing for property tuning depending on the design.

## Shaping an Amorphous Polymer

**Figure 5:**
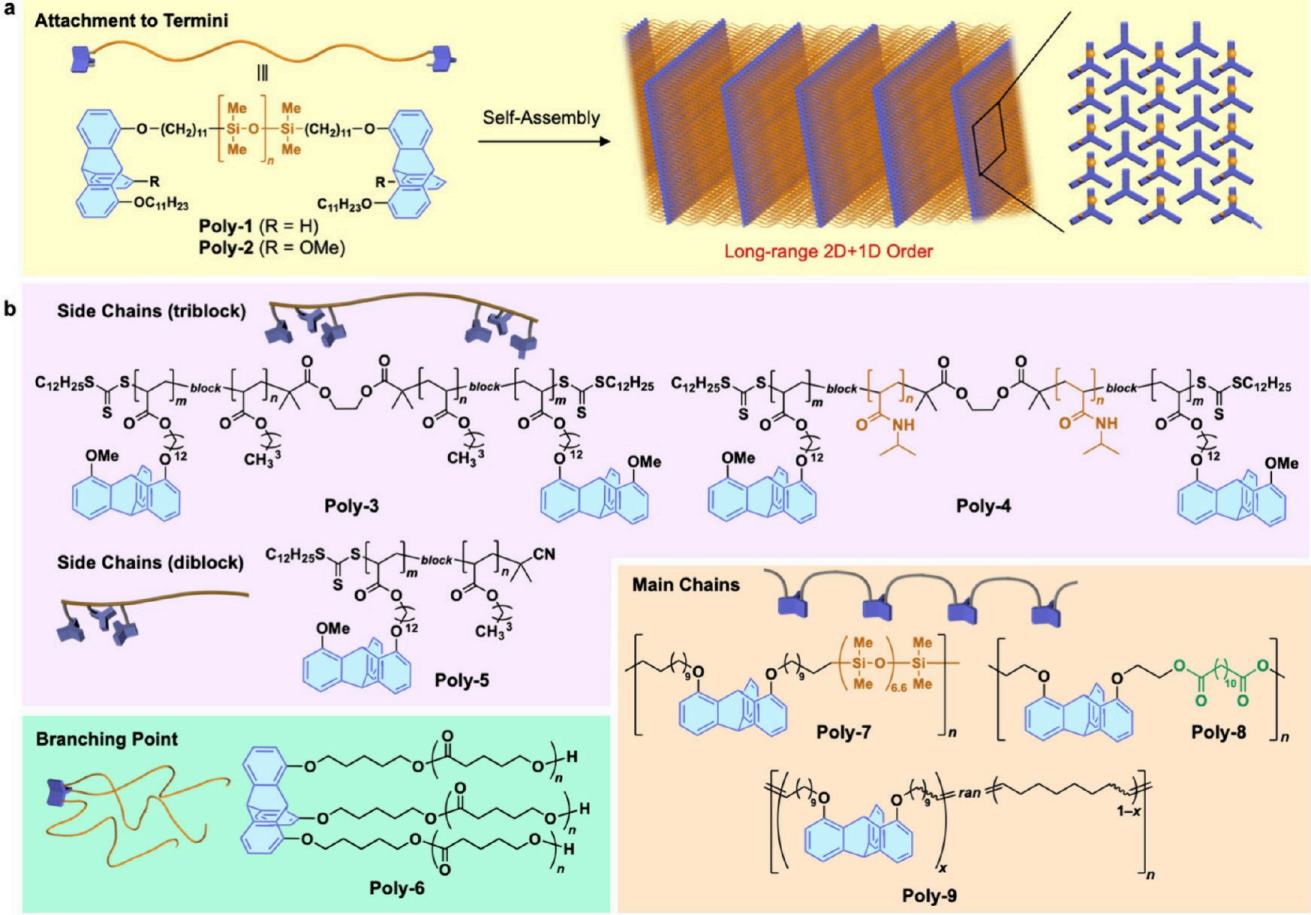
(a) Schematics of molecular and assembly structures of PDMS bearing 1,8- or 1,8,13-substituted triptycene termini (**poly-1** and **poly-2**). (b) Examples of other polymers capable of self-assembling into ordered 2D+1D structures, which contain triptycene units at the side chains (**poly-3** ∼ **poly-5**), branching point (**poly-6**), or main chains (**poly-7** ∼ **poly-9**). Adapted from ref (36) (panel a). Copyright 2018 American Chemical Society.

It has been shown that 1,8- and 1,8,13-substituted triptycenes, when incorporated into polymers even at low content levels, can promote polymer ordering into 2D+1D structures, thereby leading to significant improvements in mechanical and rheological properties.^31^ Examples include terminal functionalization for poly(dimethylsiloxane) (PDMS; **poly-1**(34) and **poly-2**(35)) (Figure 5a), attachment to acrylic polymers as their side chain components (**poly-3** ∼ **poly-5**),^36^ use as the branch point of a polyester-based three-armed polymer (**poly-6**),^37^ and incorporation into the main chains of various polymers (**poly-7** ∼ **poly-9**)^38^ (Figure 5b). The structuring of the polymers induced by the triptycene units is dramatic, and of particular interest, PDMS, which is originally a liquid polymer, changes into a solid substance in **poly-2**, exhibiting thermoplastic properties without any chemical cross-linking. This polymer also displays self-healing ability. Further details can be found in our recent review paper.^31^

## Enhancing Performance of Organic Devices

**Figure 6:**
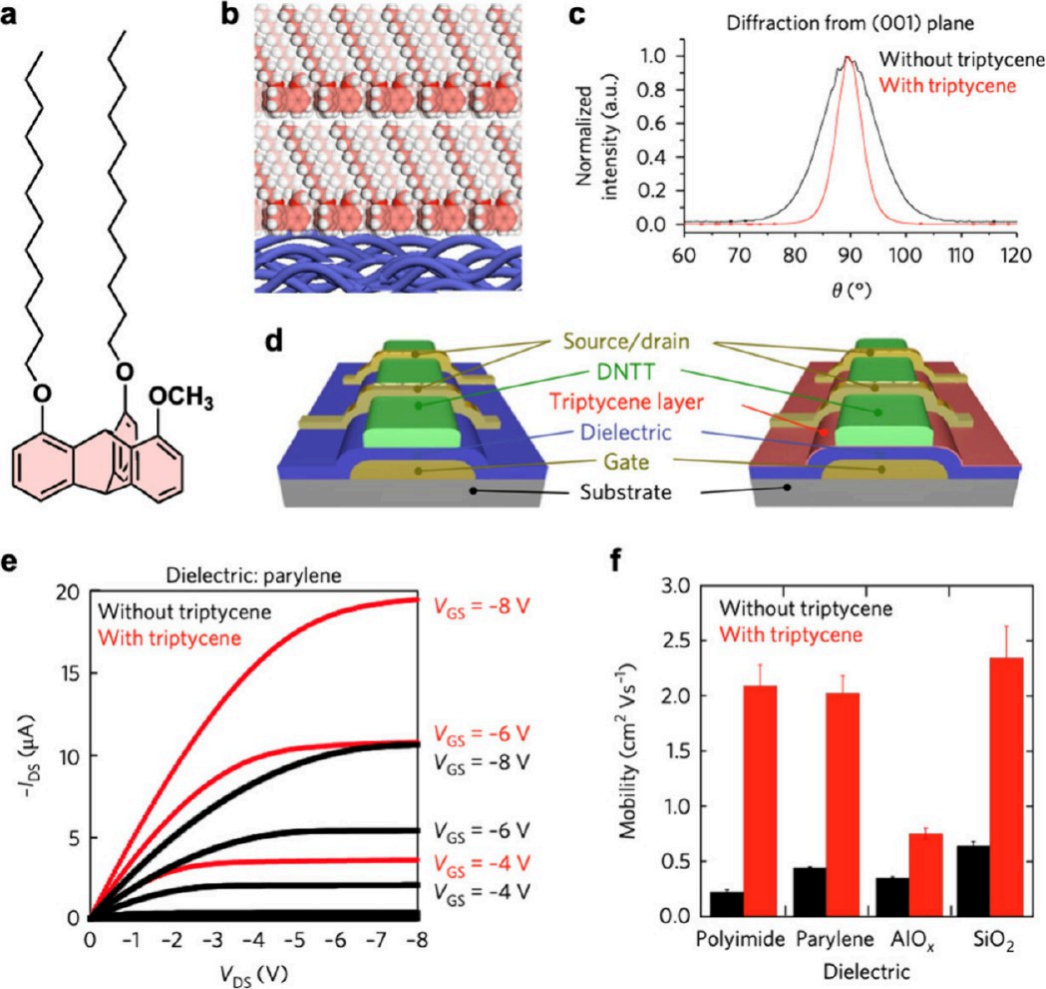
(a) Molecular structure of a tripodal triptycene used for the fabrication of OTFTs. (b) Schematic illustration of a *ca*. 5 nm-thick oriented triptycene film on a polymer substrate. (c) Angular dependence of the peak intensity for the diffraction from the (001) plane of an evaporated DNTT layer on parylene with and without a triptycene interlayer. (d) Schematic structure of OTFTs with and without a triptycene interlayer, (e) output curves for these OTFTs, and (f) mobilities of several OTFTs with different dielectrics. Adapted with permission from ref (39). Copyright 2018 Springer Nature.

Molecular films formed by paraffinic tripodal triptycenes, such as the molecule shown in Figure 6a, are useful in organic electronics.^18,39,40^ Due to their substrate-independent assembling ability, these triptycenes can form highly oriented thin films (Figure 6b). For example, when an approximately 5 nm layer of triptycene film is deposited on top of a parylene dielectric layer, followed by deposition of dinaphtho[2,3-*b*:2′,3′-*f*]thieno[3,2-*b*]thiophene (DNTT, a typical organic semiconductor), the overall performance of the resulting organic thin-film transistor (OTFT) is greatly improved compared to the case without using the triptycene film. GI-XRD data for the respective samples (Figure 6c) points to a strongly improved DNTT crystallinity in the presence of the triptycene film. A comparison of the performance of OTFTs with and without the triptycene film (Figure 6d) shows the distinct effect of this device engineering. The enhanced performance is reflected in all the OTFT characteristics, including the output curves (Figure 6e) and mobility (Figure 6f). These engineered transistors enable the fabrication of high-performance organic complementary circuits on polymer substrates, with high oscillation speeds and low operation voltages.^39^

## Tripodal Adsorption on Au(111)

**Figure 7:**
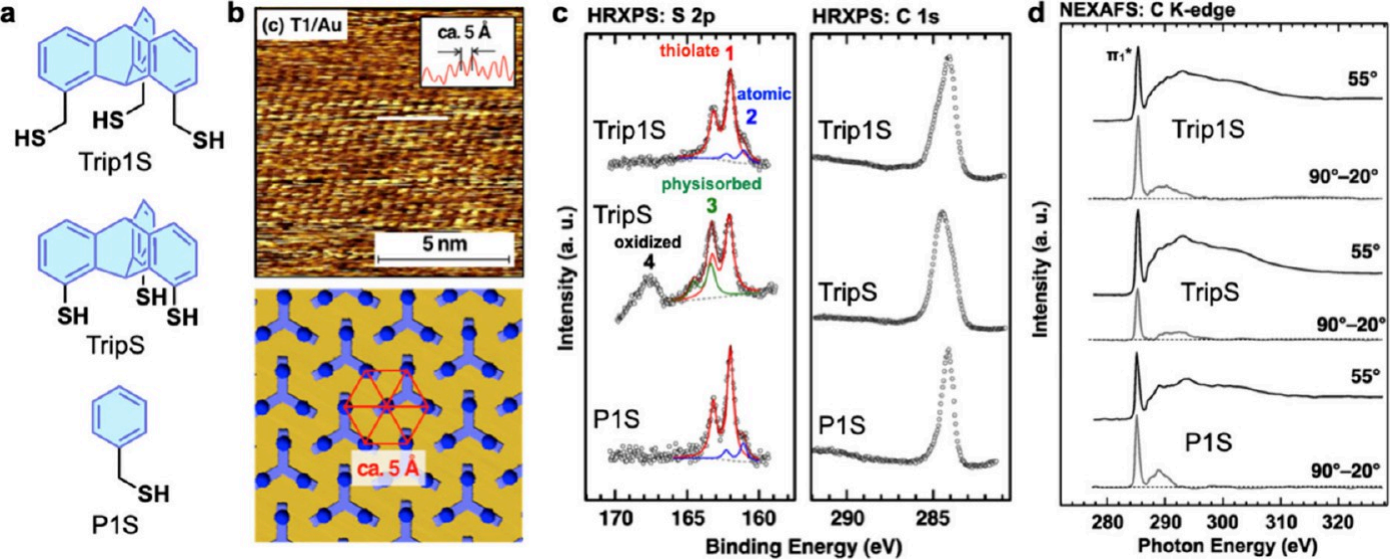
(a) SAM-forming molecules. (b) STM image of Trip1S/Au along with the derived molecular packing structure. (c) C 1s and S 2p XPS spectra of the SAMs. (d) C K-edge NEXAFS spectra of the SAMs. The spectra of Trip1S/Au exhibit particularly strong linear dichroism highlighted by the signal at the π_1_* resonance position in the 90°–20° spectra. Adapted from ref (3). Copyright 2019 American Chemical Society.

As mentioned in the Introduction, along with multilayer films that are decoupled from the substrates, tripodal-anchored, triptycene-based SAMs have been targeted. First, focusing on the thiolate anchoring group (AG) suitable for gold substrates, a typical electrode material in organic and molecular electronics,^3,41^ two types of tripods were synthesized (Figure 7a). The difference between Trip1S and TripS is the presence or absence of a methylene group in the anchoring “legs”. The SAM-forming ability of these triptycenes was compared to that of a reference monopodal compound, P1S (Figure 7a). Using the standard immersion preparation procedure, Trip1S and TripS were adsorbed on Au(111) with nested hexagonal packing (Figure 7b). Both films have monolayer thicknesses, according to the C 1s X-ray photoelectron spectroscopy (XPS) data (Figure 7c). However, only Trip1S/Au shows the sole signature of the thiolate anchoring in the S 2p spectra, similar to the monopodal case (P1S/Au), whereas TripS/Au exhibits the additional signals of physisorbed and oxidized sulfur (Figure 7c). This result suggests tripodal anchoring of Trip1S to the substrate, which is corroborated by near-edge X-ray absorption fine structure (NEXAFS) data (Figure 7d) implying, in contrast to TripS/Au, high orientational order and an almost upright orientation of the phenylene rings in Trip1S/Au. The superior properties of Trip1S/Au are attributed to the conformationally flexible methylene linkers at the anchoring groups, absent in the case of TripS. Due to this flexibility, the nested packing structure of the Trip1S monolayer can adapt to the Au(111) template, resulting in long-range structural order and a consistent tripodal adsorption configuration.

## Polymorphism and Hidden Chirality on Ag(111)

**Figure 8:**
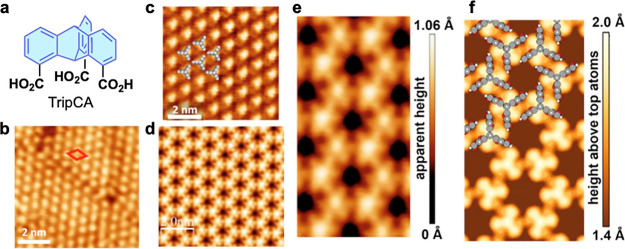
(a) Molecular structure of TripCA. STM images of (b) the nested hexagonal packing structure, (c) relaxed hexagonal structure, and (d) honeycomb structure. (e) Enlarged view of the latter structure and (f) its theoretical simulation. Proposed molecular structures are shown in (c) and (f). Reproduced from ref (19). Available under a CC-BY 4.0 license. Copyright 2021 The Authors.

Successful preparation of the tripodal triptycene SAMs on Au(111) implies that such films can also be prepared on other substrates. To this end, silver, also important for organic electronics, was examined.^19^ As the AG, carboxylic acid (TripCA), having a good affinity to silver, was selected (Figure 8a). Surprisingly, not one but several different structures were observed, depending on the preparation conditions. Besides the typical, nested hexagonal molecular arrangement (Figure 8b), a relaxed hexagonal arrangement (Figure 8c), and a honeycomb structure (Figure 8d) emerged. The latter structure is particularly intriguing as such an open arrangement is unusual for SAMs consisting of upright-standing molecules. This orientation is deduced from the dedicated spectroscopic experiments, revealing that equivalent bonding of all carboxylic acid anchoring groups in the same bidentate fashion occurs.

The honeycomb structure can only be rationalized by assuming a chiral arrangement of the molecules (Figure 8f). The seeming discrepancy between the appearance of this structure, with no obvious fingerprints of chirality, and the proposed arrangement has been resolved by density functional theory calculations, considering the actual electronic structure of the adsorbed layer and reproducing in full the STM images (Figure 8e,f). The formation of this structure, featuring a variation of the nested motif, stems presumably from a delicate balance of intermolecular interactions and the binding energy hypersurface associated with the given anchoring groups and substrate.

## Tripodal SAMs on Oxide Substrates

**Figure 9:**
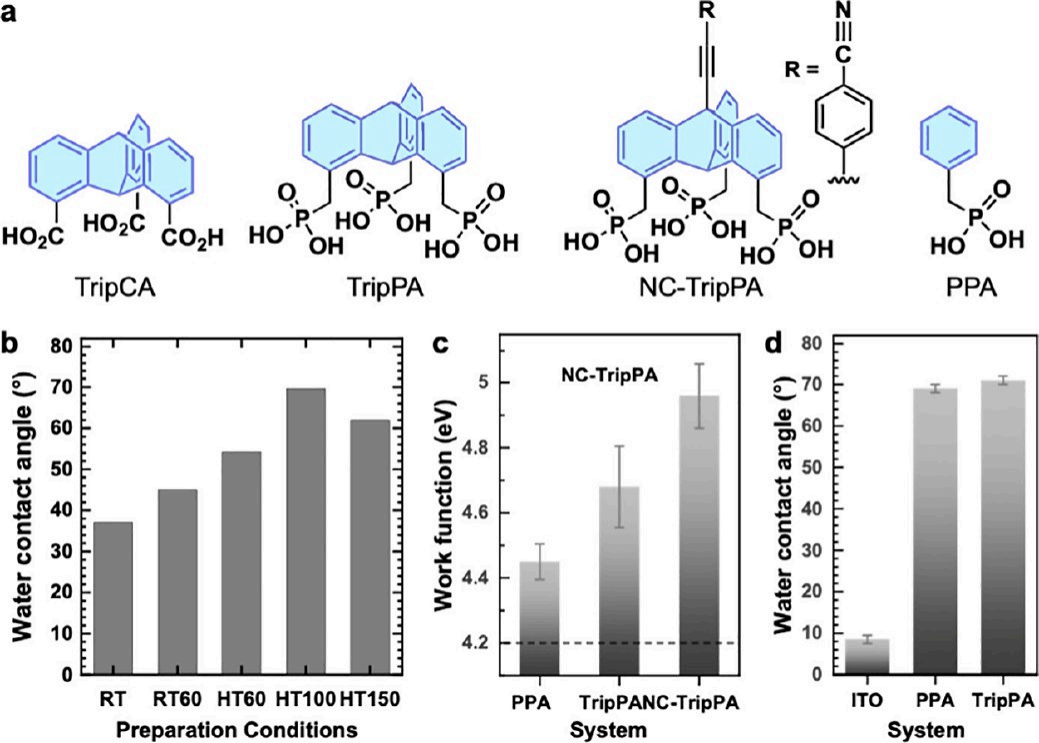
(a) SAM-forming molecules for oxide substrates. (b) WCA of TripCA/ITO at different preparation conditions. (c) WCA and (d) WF of PPA/ITO, TripPA/ITO, and NC-TripPA/ITO. (b) Adapted from ref (42). Copyrights 2023 American Chemical Society. (c, d) Adapted from ref (25). Published (2024) by the Royal Society of Chemistry under CC BY license.

Along with metal substrates that serve as electrode materials in electronic devices, oxide supports are also important. As a representative material in this context, we focus on indium tin oxide (ITO), which is frequently used as an electrode material for organic and perovskite solar cells. Here, TripCA and a tripod molecule with phosphonic acid anchors (TripPA) were used to prepare SAMs (Figure 9a).^25,42^ These SAMs exhibit dense molecular packing with similar parameters and properties to the monopodal reference monolayer with a phosphonic acid anchor (PPA). The orientational order in the SAMs of TripCA and TripPA is somewhat lower compared to the aforementioned monolayers on Au and Ag, but still sufficiently high.

In addition to extensive spectroscopic characterizations, the water contact angle (WCA) and work function (WF) of the triptycene SAMs on ITO were measured as representative fingerprint parameters. For TripCA/ITO, the preparation conditions were varied, and the optimal quality, indicated by the highest WCA (Figure 9b; compare to the blank substrate (ITO) value in Figure 9c), is achieved by employing an elevated immersion temperature and postannealing at 100 °C. A similar WCA value, even higher than that of the reference PPA monolayer, is achieved for TripPA/ITO (Figure 9c). The WF of TripPA/ITO is higher than that of PPA/ITO, and functionalization of the outermost surface of the TripPA SAM with the dipolar cyano (NC) group (NC-TripPA) results in a further increase in WF (Figure 9d), emphasizing the potential of triptycene SAMs for electrostatic engineering of oxide substrates.

## Charge Transfer Dynamics

**Figure 10:**
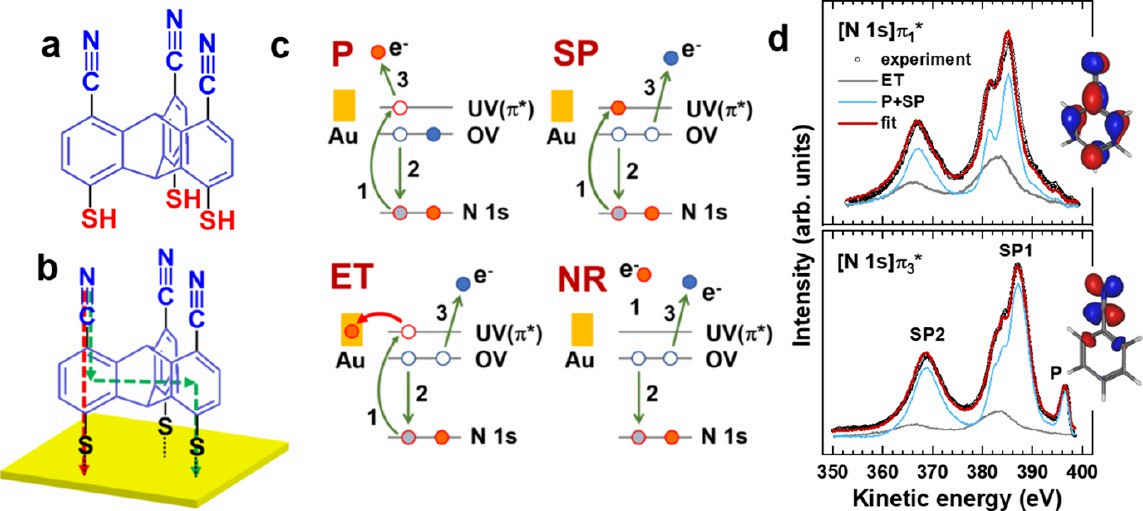
(a) Structure of NC-TripS. (b) Alternative ET pathways across the molecular framework. (c) Schematic of the relevant electronic processes. (d) [N 1s]π_1_* and [N 1s]π_3_* RAES spectra of NC-TripS/Au decomposed into individual contributions; the relevant orbitals are shown.^48^ Adapted with permission from refs (20) and (48). Copyrights 2021 American Chemical Society (ref (20)) and 2011 (ref (48)) American Physical Society.

Besides nonsubstituted triptycene SAMs, monolayers decorated with different tail groups were designed and studied in various contexts. In particular, cyano group-substituted triptycene (NC-TripS, Figure 10a) features two alternative electron transfer (ET) pathways from the tail groups to the substrate when assembled as a SAM (Figure 10b). The efficiency of these pathways, mimicking intramolecular and intermolecular ET pathways in SAMs,^43^ was tested by resonant Auger electron spectroscopy (RAES) following the core hole clock approach.^44,45^

After the excitation of an N 1s electron into a π* orbital of the cyano group, excited state decay can occur according to the standard participant (P) and spectator (SP) scenarios (Figure 10b).^46^ Alternatively, an electron can transfer to the substrate across the molecular backbone (ET), resulting in a similar final state as for nonresonant AES (NR) (Figure 10b). The P, SP, and ET processes can be distinguished by the characteristic spectra contributing to the overall RAES envelope.^46^ The lifetime of the excited state (6.4 fs for N 1s)^47^ and the relative weight of the ET component define the ET time to the substrate, τ_ET_. The RAES spectra of NC-TripS/Au presented in Figure 10c were collected for excitation into the conjugated (π_1_*) and nonconjugated (π_3_*) orbitals of the cyanobenzene blades of NC-TripS. The respective τ_ET_ values, 11.5 ± 3 and 28.5 ± 4 fs,^20^ are very close to those of the monopodal reference, NC-(C_6_H_4_)-S/Au, namely, 9.3 ± 3 and 31.5 ± 4 fs.^48^ This suggests that communication between the tail groups and the substrate in NC-TripS/Au occurs through individual blades of the adsorbed molecules and is hardly affected by the presence of the aliphatic bridge connecting these blades.

In addition, it has been demonstrated that engineering of the Au(111) substrate with the NC-TripS SAMs, bearing polar cyano groups, results in a noticeable WF increase compared to the nonpolar monolayers,^20^ in agreement with the results for NC-TripPA/ITO.^25^

## Molecular Electronics

**Figure 11:**
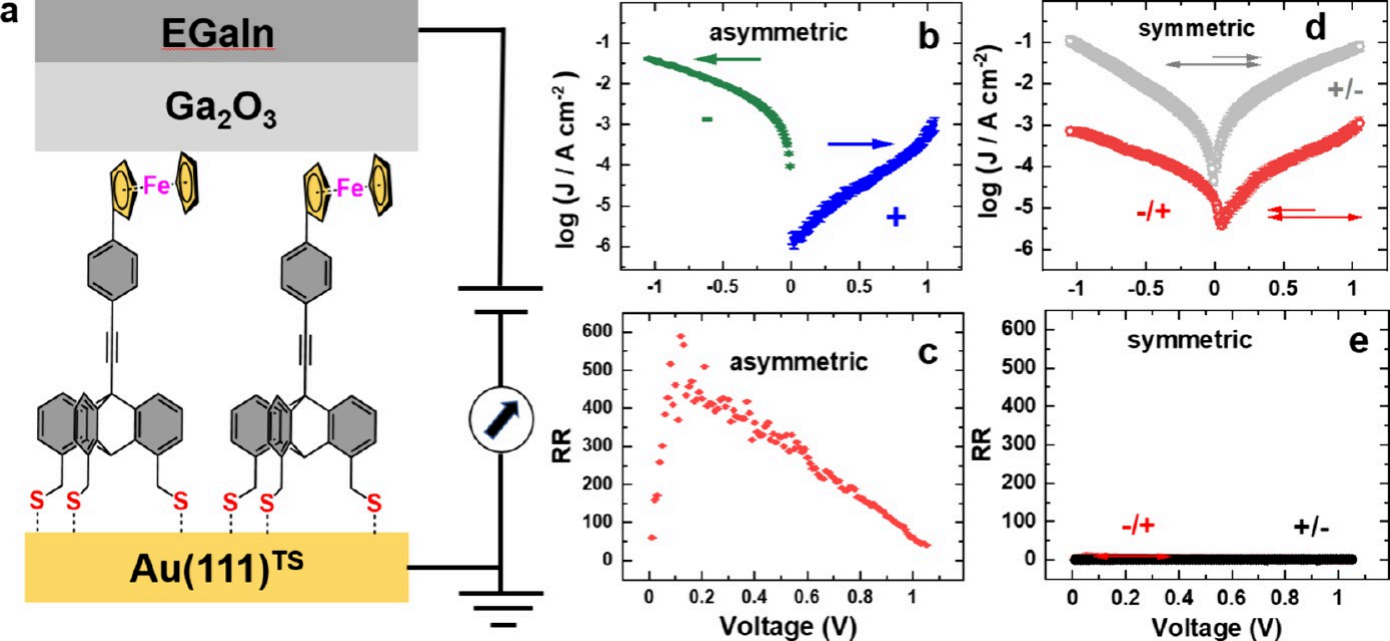
(a) Schematic structure of a two-terminal junction containing a Fc-Trip1S SAM. (b, d) *J*–*V* curves upon (b) asymmetric and (d) symmetric bias sweeping. (c, e) The respective RRs. Adapted from ref (49). Copyright 2023 American Chemical Society.

Specifically substituted triptycenes were also designed in the context of molecular electronics. A SAM of ferrocene-substituted triptycene (Fc-Trip1S), embedded into a two-terminal junction with bottom gold and top eutectic GaIn electrodes (Figure 11a), exhibits strongly asymmetric *J*-*V* curves upon asymmetric bias sweeping (Figure 11b), corresponding to a rectification factor (RR) of ∼400–600 at a very low bias of 0.1 V.^49^ However, this asymmetry disappears completely upon subsequent symmetric bias sweeping (Figure 11d), resulting in a lack of rectification (Figure 11e) and a strong difference in the *J*–*V* curves depending on the direction of the initial sweep (Figure 11d). These results are explained by the occurrence of two different conduction states, high and low. The transition between these states is mediated by bias-induced, nonreversible oxidation of the ferrocene (Fc) groups in combination with conformational changes in the molecular film.^50,51^ These results demonstrate that redox groups in molecular systems can exhibit complex behavior that is only perceivable by variation of the sweeping mode.

**Figure 12:**
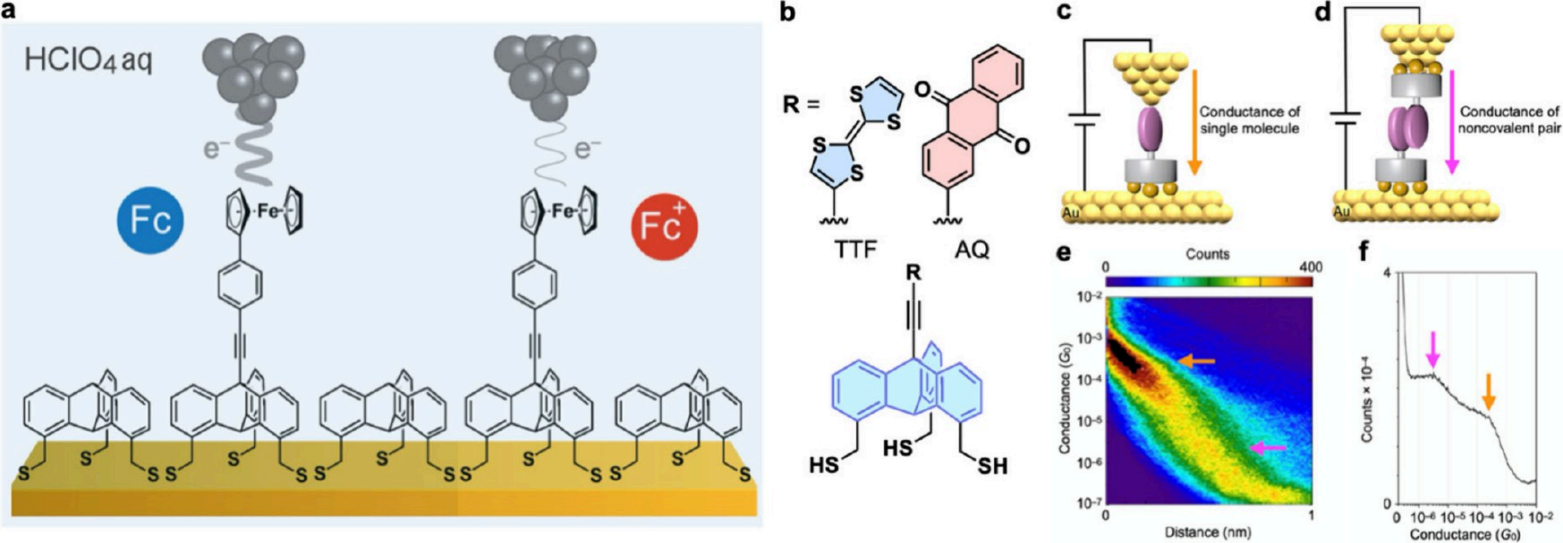
(a) Schematic illustration of EC-STM experiments to investigate the oxidation behavior of the terminal Fc group on Fc-Trip1S embedded into a Trip1S SAM matrix. (b) Molecular structures of TTF- or AQ-appended triptycene. (c, d) Models of molecular junctions. (e) 2D histogram of conductance versus stretching distance traces and (f) conductance histogram for the junction of TTF-appended triptycene as an example; the orange and purple arrows show the conductance arising from a single molecule and noncovalent pair, respectively. Adapted with permission from refs (a) (22) and (b–d) (21). Copyrights 2023 American Chemical Society (ref (22)) and 2023 The Authors; available under CC-BY-NC-ND 4.0 license (ref (21)).

Applying an electrochemical STM (EC-STM) technique to mixed Fc-Trip1S/Trip1S SAMs on Au, the redox reaction involving the Fc group on Fc-Trip1S was examined at the single-molecular level (Figure 12a).^22^ The structurally rigid Fc-Trip1S molecules were isolated in a Trip1S SAM matrix and addressed by an EC-STM tip. This methodology paves the way for versatile single-molecule measurements of important phenomena at the solid–liquid interface, such as those relevant to photochemistry and heterogeneous catalysis. As a further extension of the electrochemical approach, electron-donating tetrathiafulvalene (TTF) and electron-accepting anthraquinone (AQ) units are incorporated into a molecular junction using a tripodal triptycene scaffold for the precise orientational control over these units in the junction (Figure 12b).^21^ The obtained conductance profiles show the existence of two types of junctions formed by the individual molecules and noncovalent dimers (Figure 12c–f). The case of noncovalent pairing at the molecular level is particularly interesting from the viewpoint of molecular electronics, also showing the potential of the triptycene scaffold to precisely direct and arrange electroactive subunits.

## Molecular Optics

**Figure 13:**
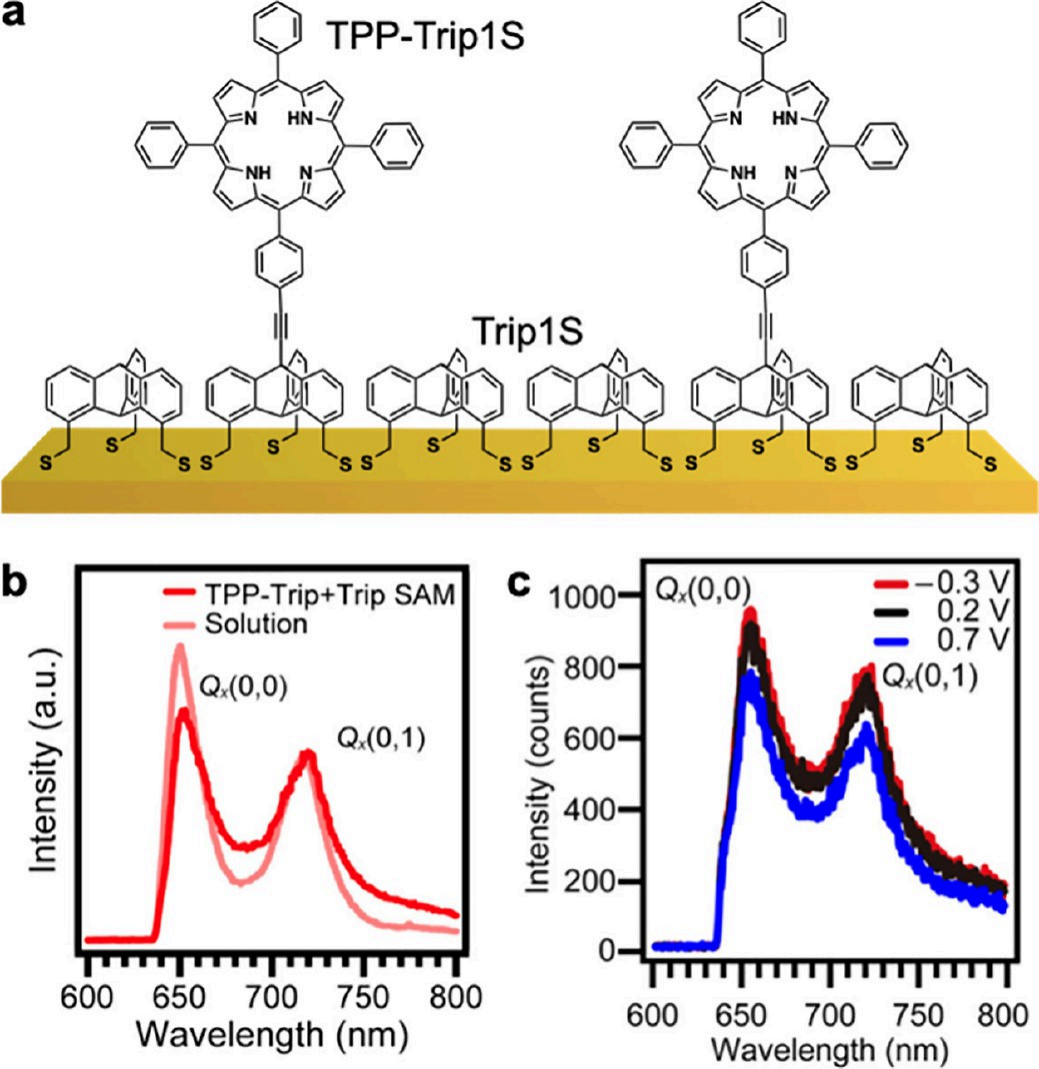
(a) Schematic illustration of TPP-Trip1S embedded into a Trip1S SAM matrix on Au(111). (b) Fluorescence spectra of the binary TPP-Trip1S/Trip1S SAMs compared to the spectra of TPP-Trip1S in solution. (c) Fluorescence spectra of the binary TPP-Trip1S/Trip1S SAMs measured under an electrochemical environment at different applied potentials. Adapted from ref (26). Copyright 2024 American Chemical Society.

The strategy of using binary SAMs can be applied to insulate optically active molecular units from metal substrates to prevent rapid quenching of their excited states. As a representative example, a tetraphenylporphyrin (TPP) dye was coupled with a triptycene scaffold and adsorbed on Au(111) together with a Trip1S SAM matrix (Figure 13a).^26^ This architecture makes the adsorption conditions of the TPP unit almost homogeneous, suppresses quenching, prevents structural changes, and reduces interactions between the TPP chromophores. Consequently, high-quality fluorescence spectra, showing the characteristic optical modes of TPP, can be recorded under different conditions, including an electrochemical environment (Figures 13b,c). In the latter case, the spectra remain nearly unchanged upon bias sweeping, apart from a certain reduction in intensity at 0.7 V, related presumably to oxidative desorption. Not only fluorescence but also absorption spectral profiles of the molecules can be recorded, underlying the versatility of this approach.

## Clickable SAM

**Figure 14:**
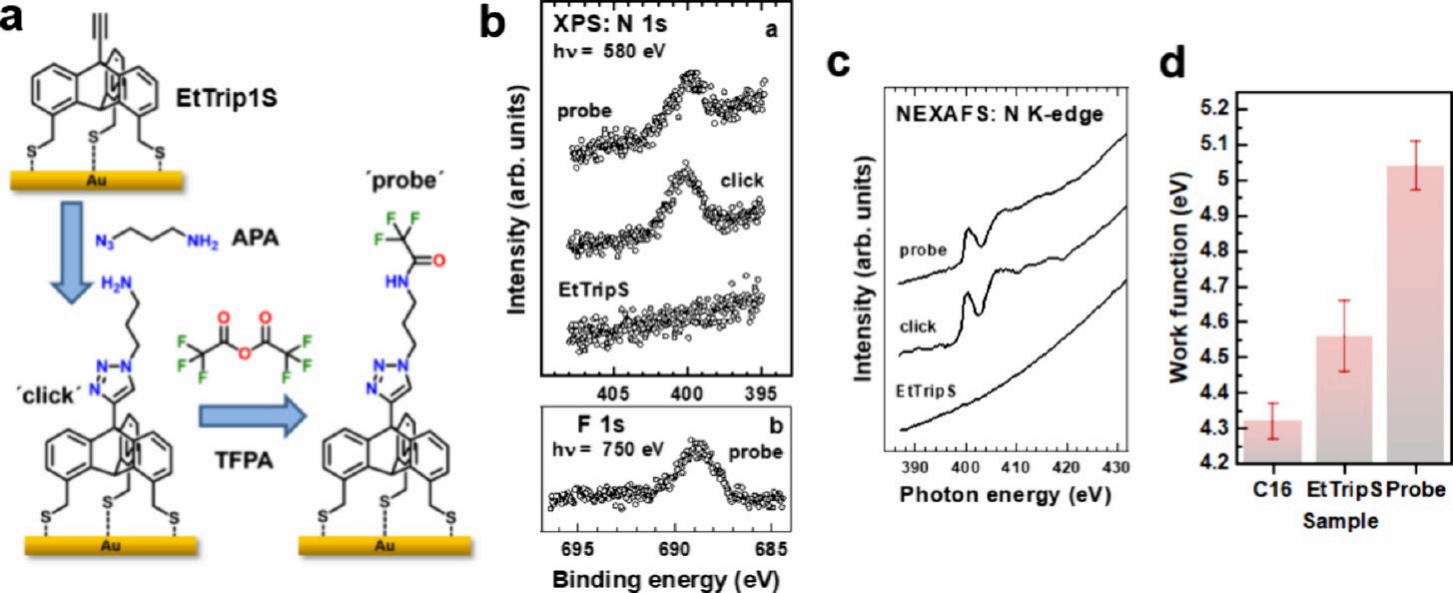
(a) Schematic illustration of the on-surface click reaction and subsequent functionalization of the EtTrip1S SAM. (b) N 1s and F 1s XPS and (c) N K-edge NEXAFS spectra of the respective samples. (d) WF values of these samples and the reference hexadecanethiolate (C16) SAM. Adapted from ref (4). Copyright 2023 American Chemical Society.

Since the synthesis of specifically substituted triptycenes is a nontrivial issue, it would be advantageous to perform the substitution after molecular assembly, using a general platform. We designed ethynyl-substituted triptycene (EtTrip1S) capable of click reaction with azide reagents (Figure 14a).^52^ In the presence of a Cu catalyst, the SAM of EtTrip1S undergoes a click reaction with 3-azido propanamine (APA), and the resulting amino-functionalized SAM can be further modified using trifluoropropanoic anhydride (TFPA), giving a trifluoromethyl-terminated SAM (Figure 14a).^4^ The respective samples show the characteristic signatures of nitrogen and fluorine in XPS (Figure 14b) and 1,2,3-triazole in NEXAFS (Figure 14c). A significant increase in WF after functionalization of the EtTrip1S SAM using TFPA over the APA linker (Figure 14d) provides the final proof of the efficiency of these click and derivatization reactions.

## Lithography and Nanomembranes

**Figure 15:**
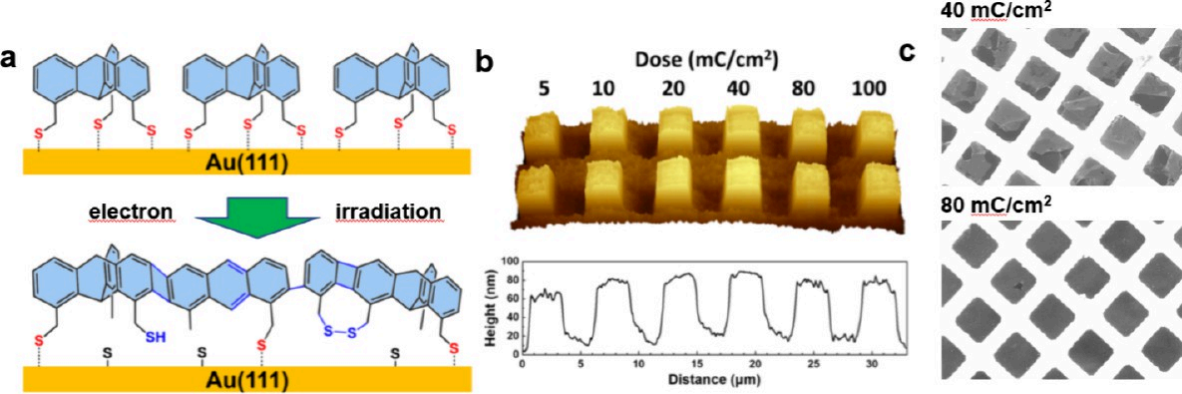
(a) Schematic illustration of the electron-irradiation-induced modification of Trip1S/Au. (b) AFM image (3D view) of Au/Si(100) patterns created using EBL (5 keV) with the Trip1S resist, along with the respective height profile across the written, square-shaped Au features. The dose values corresponding to the individual features are marked. (c) SEM images of the CNMs fabricated with two different doses. Adapted from ref (53). Copyright 2023 American Chemical Society.

The dense SAMs of tripodal triptycenes on solid supports can be used for nanofabrication. In particular, the Trip1S SAM on Au(111) is cross-linked by electron irradiation (Figure 15a),^53^ similar to monopodal aromatic monolayers.^54^ The cross-linking protects the underlying substrate from etching, proving the suitability of the Trip1S SAM as a negative resist in electron beam lithography (EBL). A representative lithographic pattern, obtained after etching of an EBL-processed Trip1S/Au template, is shown in Figure 15b, along with the height profile across the written features. The written, square-shaped pattern is adequately transferred to the Au substrate in the entire range of the applied electron doses.

The extensively cross-linked Trip1S SAM can also be separated from the substrate forming a robust and defect-free carbon nanomembrane (CNM). The electron dose required for efficient cross-linking (80 mC/cm^2^) is however higher than that for conventional aromatic SAMs (40–50 mC/cm^2^)^55^ as shown by representative SEM images (Figure 15c; the top CNM shows a variety of defects). Remarkably, the Trip1S SAM features the lowest lateral material density used to date for CNM fabrication,^55^ which makes it unique in this regard.

## Concluding Remarks

As mentioned above, triptycene is a classical molecule, but depending on the position of the substituents, it provides a versatile molecular building block that expands possibilities in various applications. The basic structure of triptycene discussed here has functional groups at the 1,8,13-positions, in contrast to many triptycene derivatives that have been synthesized so far, which have functional groups on the sides of the molecular backbone. Since assemblies of this type of triptycene, *i*.*e*., tripodal triptycene, are characterized by a 2D+1D structure, they are particularly useful for the formation of multilayer thin films and monolayers on substrate surfaces as well as for interface engineering, in addition to self-assembly in the bulk state. In the context of multilayers, high structural and orientational perfection and the possibility to integrate functional molecular units are probably the most important features. In the context of monolayers, robust tripodal adsorption configurations, adaptivity to different substrate types, molecular-level control of orientation and electrochemistry, and the possibility of on-surface functionalization are the most favorable properties. The potential of these systems is already well-recognized by the community^30,31,56−61^ but we hope that the present Account will attract more researchers to the given field and lead to a strong impulse for new studies and applications of tripodal triptycenes in various areas.

## References

[ref1] Seiki N.; Shoji Y.; Kajitani T.; Ishiwari F.; Kosaka A.; Hikima T.; Takata M.; Someya T.; Fukushima T. Rational Synthesis of Organic Thin Films with Exceptional Long-Range Structural Integrity. Science 2015, 348, 1122–1126. 10.1126/science.aab1391. 26045433

[ref2] Leung F. K.-C.; Ishiwari F.; Kajitani T.; Shoji Y.; Hikima T.; Takata M.; Saeki A.; Seki S.; Yamada Y. M. A.; Fukushima T. Supramolecular Scaffold for Tailoring the Two-Dimensional Assembly of Functional Molecular Units into Organic Thin Films. J. Am. Chem. Soc. 2016, 138, 11727–11733. 10.1021/jacs.6b05513. 27549349

[ref3] Ishiwari F.; Nascimbeni G.; Sauter E.; Tago H.; Shoji Y.; Fujii S.; Kiguchi M.; Tada T.; Zharnikov M.; Zojer E.; Fukushima T. Triptycene Tripods for the Formation of Highly Uniform and Densely Packed Self-Assembled Monolayers with Controlled Molecular Orientation. J. Am. Chem. Soc. 2019, 141, 5995–6005. 10.1021/jacs.9b00950. 30869881 PMC6483319

[ref4] Das S.; Ishiwari F.; Shoji Y.; Fukushima T.; Zharnikov M. Triptycene-Based Self-Assembled Monolayer as a Template for Successive Click Reactions. J. Phys. Chem. C 2023, 127, 5178–5185. 10.1021/acs.jpcc.3c00443.

[ref5] Ishiwari F.; Shoji Y.; Fukushima T. Supramolecular Scaffolds Enabling the Controlled Assembly of Functional Molecular Units. Chem. Sci. 2018, 9, 2028–2041. 10.1039/C7SC04340F. 29719683 PMC5896469

[ref6] Marc Veen E.; Feringa B. L.; Postma P. M.; Jonkman H. T.; Spek A. L. Solid State Organisation of C60 by Inclusion Crystallisation with Triptycenes. Chem. Commun. 1999, 1709–1710. 10.1039/a904457d.

[ref7] Konarev D. V.; Khasanov S. S.; Otsuka A.; Maesato M.; Saito G.; Lyubovskaya R. N. A Two-Dimensional Organic Metal Based on Fullerene. Angew. Chem., Int. Ed. 2010, 49, 4829–4832. 10.1002/anie.201001463. 20533484

[ref8] Schreiber F. Self-Assembled Monolayers: From ‘Simple’ Model Systems to Biofunctionalized Interfaces. J. Phys.: Condens. Matter 2004, 16, R881–R900. 10.1088/0953-8984/16/28/R01.

[ref9] Love J. C.; Estroff L. A.; Kriebel J. K.; Nuzzo R. G.; Whitesides G. M. Self-assembled monolayers of thiolates on metals as a form of nanotechnology. Chem. Rev. 2005, 105, 1103–1169. 10.1021/cr0300789. 15826011

[ref10] Kind M.; Woll C. Organic Surfaces Exposed by Self-Assembled Organothiol Monolayers: Preparation, Characterization, and Application. Prog. Surf. Sci. 2009, 84, 230–278. 10.1016/j.progsurf.2009.06.001.

[ref11] Vericat C.; Vela M. E.; Benitez G.; Carro P.; Salvarezza R. C. Self-Assembled Monolayers of Thiols and Dithiols on Gold: New Challenges for a Well-Known System. Chem. Soc. Rev. 2010, 39, 1805–1834. 10.1039/b907301a. 20419220

[ref12] Chinwangso P.; Jamison A. C.; Lee T. R. Multidentate Adsorbates for Self-Assembled Monolayer Films. Acc. Chem. Res. 2011, 44, 511–519. 10.1021/ar200020s. 21612198

[ref13] Valášek M.; Lindner M.; Mayor M. Rigid Multipodal Platforms for Metal Surfaces. Beilstein J. Nanotechnol. 2016, 7, 374–405. 10.3762/bjnano.7.34. 27335731 PMC4901557

[ref14] Valasek M.; Mayor M. Spatial and Lateral Control of Functionality by Rigid Molecular Platforms. Chem. Eur. J. 2017, 23, 13538–13548. 10.1002/chem.201703349. 28766790

[ref15] Li Z.-Q.; Tang J.-H.; Zhong Y.-W. Multidentate Anchors for Surface Functionalization. Chem. Asian J. 2019, 14, 3119–3126. 10.1002/asia.201900989. 31389657

[ref16] Shioya H.; Shoji Y.; Seiki N.; Nakano M.; Fukushima T.; Iwasa Y. Raising the Metal–Insulator Transition Temperature of VO_2_ Thin Films by Surface Adsorption of Organic Polar Molecules. Appl. Phys. Express 2015, 8, 121101 10.7567/APEX.8.121101.

[ref17] Kumano M.; Ide M.; Seiki N.; Shoji Y.; Fukushima T.; Saeki A. A Ternary Blend of a Polymer, Fullerene, and Insulating Self-Assembling Triptycene Molecules for Organic Photovolatics. J. Mater. Chem. A 2016, 4, 18490–18498. 10.1039/C6TA07705F.

[ref18] Kondo M.; Kajitani T.; Uemura T.; Noda Y.; Ishiwari F.; Shoji Y.; Araki T.; Yoshimoto S.; Fukushima T.; Sekitani T. Highly-Ordered Triptycene Modifier Layer Based on Blade Coating for Ultraflexible Organic Transistors. Sci. Rep. 2019, 9, 9200 10.1038/s41598-019-45559-4. 31235730 PMC6591239

[ref19] Das S.; Nascimbeni G.; de la Morena R. O.; Ishiwari F.; Shoji Y.; Fukushima T.; Buck M.; Zojer E.; Zharnikov M. Porous Honeycomb Self-Assembled Monolayers: Tripodal Adsorption and Hidden Chirality of Carboxylate Anchored Triptycenes on Ag. ACS Nano 2021, 15, 11168–11179. 10.1021/acsnano.1c03626. 34125529 PMC8320238

[ref20] Das S.; Asyuda A.; Shoji Y.; Kosaka A.; Fukushima T.; Zharnikov M. Cyano-Substituted Triptycene-Based Monolayers on Au(111): Tripodal Adsorption, Dipole Engineering, and Charge Transfer. J. Phys. Chem. C 2021, 125, 18968–18978. 10.1021/acs.jpcc.1c05618.

[ref21] Martin C. J.; Fukui T.; Takehara R.; Fujii S.; Fukushima T. Precise Orientational Control of Electroactive Units Using a Tripodal Triptycene Scaffold to Direct Noncovalent Pairing at the Single Molecular Level. Precis. Chem. 2023, 1, 388–394. 10.1021/prechem.3c00070. PMC12382406 40880976

[ref22] Kobayashi Y.; Yokota Y.; Wong R. A.; Hong M.; Takeya J.; Osawa S.; Ishiwari F.; Shoji Y.; Harimoto T.; Sugimoto K.; et al. Single-Molecule Observation of Redox Reactions Enabled by Rigid and Isolated Tripodal Molecules. J. Phys. Chem. C 2023, 127, 746–758. 10.1021/acs.jpcc.2c07362.

[ref23] Fukui T.; Hofuku K.; Kosaka A.; Minoi N.; Nishikubo R.; Ishiwari F.; Sato H.; Saeki A.; Fukushima T. Triammonium Molecular Tripods as Organic Building Blocks for Hybrid Perovskite Solar Cells. Small Struct. 2024, 5, 2300411 10.1002/sstr.202300411.

[ref24] Mizoue R.; Takeda T.; Dekura S.; Kato M.; Fukui T.; Shoji Y.; Fukushima T.; Yamane S.; Suzuki Y.; Kawamata J.; Akutagawa T. Ferroelectricity of alkylamide-substituted triptycene derivatives. J. Mater. Chem. C 2024, 12, 5578–5586. 10.1039/D3TC04752K.

[ref25] Zhang C.; Das S.; Sakurai N.; Imaizumi T.; Sanjayan S.; Shoji Y.; Fukushima T.; Zharnikov M. Phosphonic Acid Anchored Tripodal Molecular Films on Indium Tin Oxide. Phys. Chem. Chem. Phys. 2024, 26, 11360–11369. 10.1039/D4CP00892H. 38567399

[ref26] Kobayashi Y.; Yokota Y.; Shoji Y.; Sajisha S.; Martin C. J.; Takeya J.; Fukushima T.; Kim Y. Fluorescence Detection of Tetraphenylporphyrin Isolated on the Au(111) Electrode Enabled by Tripodal Molecules. J. Phys. Chem. C 2024, 128, 15082–15090. 10.1021/acs.jpcc.4c04400.

[ref27] Ogawa T.; Ishiwari F.; Hajjaj F.; Shoji Y.; Kajitani T.; Yazawa K.; Ohkubo T.; Fukushima T. 2D Hexagonal Assembly of a Dipolar Rotor with a Close Interval of 0.8 nm Using a Triptycene-based Supramolecular Scaffold. Chem. Sci. 2024, 15, 11021–11028. 10.1039/D4SC02750G. 39027311 PMC11253181

[ref28] Fukumitsu M.; Fukui T.; Shoji Y.; Kajitani T.; Khan R.; Tkachenko N. V.; Sakai H.; Hasobe T.; Fukushima T. Supramolecular Scaffold-Directed Two-Dimensional Assembly of Pentacene into a Configuration to Facilitate Singlet Fission. Sci. Adv. 2024, 10, eadn7763 10.1126/sciadv.adn7763. 39270030 PMC11397492

[ref29] Iwata T.; Shindo M. Synthesis of 1,8,13-Substituted Triptycenes. Chem. Lett. 2021, 50, 39–51. 10.1246/cl.200600.

[ref30] Bastien G.; Severa L.; Škuta M.; Santos Hurtado C.; Rybaček J.; Šolinova V.; Cisařova I.; Kašička V.; Kaleta J. Triptycene-Based Tripodal Molecular Platforms. Chem. Eur. J. 2024, e202401889 10.1002/chem.202401889. 39282809

[ref31] Ishiwari F.; Shoji Y.; Martin C. J.; Fukushima T. Recent Advances in Structurally Elaborate Triptycenes, Triptycene-Containing Polymers and Assemblies: Structures, Functions and Applications. Polym. J. 2024, 56, 791–818. 10.1038/s41428-024-00920-x.

[ref32] Hazell R. G.; Pawley G. S.; Lund Petersen C. E. Crystal and Molecular Structure of Triptycene, Refined Using Constraint Procedures. J. Cryst. Mol. Struct. 1971, 1, 319–324. 10.1007/BF01200806.

[ref33] Xu Q.-M.; Han M.-J.; Wan L.-J.; Wang C.; Bai C.-L.; Dai B.; Yang J.-L. Tuning Molecular Orientation with STM at the Solid/Liquid Interface. Chem. Commun. 2003, 2874–2785. 10.1039/b308155a. 14680218

[ref34] Ishiwari F.; Okabe G.; Ogiwara H.; Kajitani T.; Tokita M.; Takata M.; Fukushima T. Terminal Functionalization with a Triptycene Motif that Dramatically Changes the Structural and Physical Properties of an Amorphous Polymer. J. Am. Chem. Soc. 2018, 140, 13497–13502. 10.1021/jacs.8b09242. 30281289

[ref35] Chen Y.; Ishiwari F.; Fukui T.; Kajitani T.; Liu H.; Liang X.; Nakajima K.; Tokita M.; Fukushima T. Overcoming the Entropy of Polymer Chains by Making a Plane with Terminal Groups: A Thermoplastic PDMS with a Long-Range 1D Structural Order. Chem. Sci. 2023, 14, 2431–2440. 10.1039/D2SC05491D. 36873840 PMC9977418

[ref36] Yu J.; Itagaki A.; Chen Y.; Fukui T.; Ishiwari F.; Kajitani T.; Fukushima T. Effective Design for Long-Range Polymer Ordering Using Triptycene-Containing Side Chains. Macromolecules 2023, 56, 4556–4565. 10.1021/acs.macromol.3c00795.

[ref37] Ogiwara H.; Ishiwari F.; Kimura T.; Yamashita Y.; Kajitani T.; Sugimoto A.; Tokita M.; Takata M.; Fukushima T. Changing the Structural and Physical Properties of 3-Arm Star Poly(δ-valerolactone)s by a Branch-Point Design. Chem. Commun. 2021, 57, 3901–3904. 10.1039/D1CC01092A. 33871532

[ref38] Ishiwari F.; Okabe G.; Kajitani T.; Fukushima T. Introduction of Triptycene with a Particular Substitution Pattern into Polymer Chains Can Dramatically Improve the Structural and Rheological Properties. ACS Macro Lett. 2021, 10, 1529–1534. 10.1021/acsmacrolett.1c00660. 35549132

[ref39] Yokota T.; Kajitani T.; Shidachi R.; Tokuhara T.; Kaltenbrunner M.; Shoji Y.; Ishiwari F.; Sekitani T.; Fukushima T.; Someya T. A Few-Layer Molecular Film on Polymer Substrates to Enhance the Performance of Organic Devices *Nat*. Nanotechnol. 2018, 13, 139–144. 10.1038/s41565-017-0018-6. 29255288

[ref40] Kondo M.; Uemura T.; Ishiwari F.; Kajitani T.; Shoji Y.; Morita M.; Namba N.; Inoue Y.; Noda Y.; Araki T.; Fukushima T.; Sekitani T. Ultralow-Noise Organic Transistors Based on Polymeric Gate Dielectrics with Self-Assembled Modifiers. ACS Appl. Mater. Interfaces 2019, 11, 41561–41569. 10.1021/acsami.9b13056. 31594305

[ref41] Tada T.; Ishiwari F.; Shoji Y.; Fukushima T. First-Principles Study of the Adsorption Behavior of Triptycene Molecular Tripods on Au(111): Site Selectivity and Unambiguous Molecular Orientation. J. Phys. Chem. C 2019, 123, 4401–4406. 10.1021/acs.jpcc.9b00869.

[ref42] Das S.; Ishiwari F.; Shoji Y.; Fukushima T.; Zharnikov M. Triptycene-Based Tripodal Self-Assembled Monolayer on Indium Tin Oxide. J. Phys. Chem. C 2023, 127, 2088–2097. 10.1021/acs.jpcc.2c08390.

[ref43] Slowinski K.; Chamberlain R. V.; Miller C. J.; Majda M. Through-Bond and Chain-to-Chain Coupling. Two Pathways in Electron Tunneling through Liquid Alkanethiol Monolayers on Mercury Electrodes. J. Am. Chem. Soc. 1997, 119, 11910–11919. 10.1021/ja971921l.

[ref44] Brühwiler P. A.; Karis O.; Mårtensson N. Charge-Transfer Dynamics Studied Using Resonant Core Spectroscopies. Rev. Mod. Phys. 2002, 74, 703–740. 10.1103/RevModPhys.74.703.

[ref45] Menzel D. Ultrafast Charge Transfer at Surfaces Accessed by Core Electron Spectroscopies. Chem. Soc. Rev. 2008, 37, 2212–2223. 10.1039/b719546j. 18818824

[ref46] Zharnikov M. Femtosecond Charge Transfer Dynamics in Monomolecular Films in the Context of Molecular Electronics. Acc. Chem. Res. 2020, 53, 2975–2984. 10.1021/acs.accounts.0c00627. 33232123

[ref47] Kempgens B.; Kivimäki A.; Neeb M.; Köppe H. M.; Bradshaw A. M.; Feldhaus J. A High-Resolution N1s Photoionization Study of the N_2_ Molecule in the Near-Threshold Region. J. Phys. B: At., Mol. Opt. Phys. 1996, 29, 5389–5403. 10.1088/0953-4075/29/22/016.

[ref48] Hamoudi H.; Neppl S.; Kao P.; Schüpbach B.; Feulner P.; Terfort A.; Allara D.; Zharnikov M. Orbital-Dependent Charge Transfer Dynamics in Conjugated Self-Assembled Monolayers. Phys. Rev. Lett. 2011, 107, 027801 10.1103/PhysRevLett.107.027801. 21797640

[ref49] Liu Y.; Sanjayan S.; Shoji Y.; Fukushima T.; Zharnikov M. Appearance of Different Conductance States in Monomolecular Films of Ferrocene-Decorated Triptycene-Based Tripods. J. Phys. Chem. C 2023, 127, 24458–24466. 10.1021/acs.jpcc.3c06634.

[ref50] Asyuda A.; Das S.; Lang H.; Zojer E.; Zharnikov M. Bias-Triggered Conductivity Switching and High Effective Rectification in Metallocene-Based Molecular Junctions. Adv. Electron. Mater. 2022, 8, 2200296 10.1002/aelm.202200296.

[ref51] Liu Y.; Zojer E.; Zharnikov M. Sweep-Character-Dependent Switching of the Conductance State in Ferrocene-Substituted Thiofluorene Self-Assembled Monolayers. ACS Appl. Mater. Interfaces 2022, 14, 52499–52507. 10.1021/acsami.2c15308. 36355841

[ref52] Lutz J.-F. 1,3-dipolar Cycloadditions of Azides and Alkynes: A Universal Ligation Tool in Polymer and Materials Science. Angew. Chem., Int. Ed. 2007, 46, 1018–1025. 10.1002/anie.200604050. 17211903

[ref53] Zhao Z.; Fukushima T.; Zharnikov M. Electron-Induced Modification of Triptycene Self-Assembled Monolayer in Context of Lithography and Nanofabrication. J. Phys. Chem. C 2023, 127, 15582–15590. 10.1021/acs.jpcc.3c03083.

[ref54] Angelova P.; Vieker H.; Weber N.-E.; Matei D.; Reimer O.; Meier I.; Kurasch S.; Biskupek J.; Lorbach D.; Wunderlich K.; Chen L.; Terfort A.; Klapper M.; Müllen K.; Kaiser U.; Gölzhäuser A.; Turchanin A. A Universal Scheme to Convert Aromatic Molecular Monolayers into Functional Carbon Nanomembranes. ACS Nano 2013, 7, 6489–6497. 10.1021/nn402652f. 23802686

[ref55] Turchanin A.; Gölzhäuser A. Carbon nanomembranes. Adv. Mater. 2016, 28, 6075–6103. 10.1002/adma.201506058. 27281234

[ref56] Baran Ł.; Rżysko W.; Słyk E. Simulations of the 2D Self-Assembly of Tripod-Shaped Building Blocks. Beilstein J. Nanotechnol. 2020, 11, 884–890. 10.3762/bjnano.11.73. 32566438 PMC7296195

[ref57] Kaletova E.; Santos Hurtado C.; Cisařova I.; Teat S. J.; Kaleta J. Triptycene-Based Molecular Rods for Langmuir-Blodgett Monolayers. ChemPlusChem. 2022, 87, e202200023 10.1002/cplu.202200023. 35195369

[ref58] Mistry J.-R.; Montanaro S.; Wright I. A. Homoconjugation Effects in Triptycene Based Organic Optoelectronic Materials. Mater. Adv. 2023, 4, 787–803. 10.1039/D2MA00523A.

[ref59] Liu P.; Zheng Z.; Wang H.; Wang P.; Hu Z.; Gao H.-Y. Characterize and Mediate Assembly of Triptycenes on Au(111) Surface. ACS Nano 2024, 18, 16248–16256. 10.1021/acsnano.4c02648. 38861269

[ref60] Stockerl W. J.; Reißenweber L.; Gerwien A.; Bach N. N.; Thumser S.; Mayer P.; Gschwind R. M.; Dube H. Azotriptycenes: Photoswitchable Molecular Brakes. Chem. Eur. J. 2024, 30, e202302267 10.1002/chem.202302267. 37779321

[ref61] Amin M. K.; Ye C.; Pang S.; Liu Y.; Taylor D.; Nichol G. S.; McKeown N. B. Triptycene-like Naphthopleiadene as a Readily Accessible Scaffold for Supramolecular and Materials Chemistry. Chem. Sci. 2024, 15, 14968–14976. 10.1039/D4SC02755H. 39211740 PMC11348350

